# Developing a production workflow for 3D-printed temporal bone surgical simulators

**DOI:** 10.1186/s41205-024-00218-x

**Published:** 2024-05-30

**Authors:** Andre Jing Yuen Ang, Shu Ping Chee, Joyce Zhi En Tang, Ching Yee Chan, Vanessa Yee Jueen Tan, Jordan Adele Lee, Thomas Schrepfer, Noor Mohamed Nisar Ahamed, Mark Bangwei Tan

**Affiliations:** 1 Duke-NUS Medical School, Singapore, Singapore; 2 3D Printing Centre Singapore General Hospital, Singapore, Singapore; 3 Department of Otorhinolaryngology- Head & Neck Surgery, Singapore General Hospital, Singapore, Singapore; 4 Department of Otolaryngology, KK Women’s and Children’s Hospital, Singapore, Singapore; 5 Sunshine Coast Hospital and Health Service, Sunshine Coast, Australia; 6 Department of Otolaryngology, University of Florida, Florida, USA; 7 Department of Neuroradiology & 3D Printing Centre Singapore General Hospital, Singapore, Singapore

**Keywords:** Temporal bone, Otology, 3D printing, Image segmentation, Stereolithography, Silicone molding

## Abstract

**Introduction:**

3D-printed temporal bone models enable the training and rehearsal of complex otological procedures. To date, there has been no consolidation of the literature regarding the developmental process of 3D-printed temporal bone models. A brief review of the current literature shows that many of the key surgical landmarks of the temporal bone are poorly represented in models. This study aims to propose a novel design and production workflow to produce high-fidelity 3D-printed temporal bone models for surgical simulation.

**Methods:**

Developmental phases for data extraction, 3D segmentation and Computer Aided Design (CAD), and fabrication are outlined. The design and fabrication considerations for key anatomical regions, such as the mastoid air cells and course of the facial nerve, are expounded on with the associated strategy and design methods employed. To validate the model, radiological measurements were compared and a senior otolaryngologist performed various surgical procedures on the model.

**Results:**

Measurements between the original scans and scans of the model demonstrate sub-millimetre accuracy of the model. Assessment by the senior otologist found that the model was satisfactory in simulating multiple surgical procedures.

**Conclusion:**

This study offers a systematic method for creating accurate 3D-printed temporal bone models for surgical training. Results show high accuracy and effectiveness in simulating surgical procedures, promising improved training and patient outcomes.

**Supplementary Information:**

The online version contains supplementary material available at 10.1186/s41205-024-00218-x.

## Introduction

 Surgery for temporal bone disease is challenging due to the complex operative anatomy. Traditional methods of developing surgical expertise in otolaryngology includes practice on cadaveric specimens. These specimens are associated with high costs, are limited to episodic training courses, and require specialised lab facilities to maintain. 3D printing exists as an effective method to develop temporal bone models that make training opportunities more accessible and readily enable patient-specific rehearsal of complex operations. These include procedures such as mastoidectomy, as well as more complex surgeries of the middle and inner ear such as tympanomeatal flap elevation, facial recess, and round window dissection, stapedectomy and cochlear implantation. Simulating and rehearsing these procedures necessitate temporal bone models [1–3] which depict key surgical landmarks. These include the tympanic membrane, the intraosseous facial nerve, the lateral semicircular canal as well as the ossicles. Additionally, such models should be safe for surgical drilling and cost-effective [2].

It is necessary to understand the design considerations of and develop a workflow for the production of 3D-printed temporal bones. To date there has not been a systematic review of how 3D-printed temporal bone models are designed and produced. We review the development methods described in the current literature, outline the design considerations, and propose a production workflow for 3D-printed temporal bone models.

### Literature review search terms

The systematic review was conducted in accordance with the PRISMA 2009 guidelines [4]. The MEDLINE (Pubmed) and SCOPUS databases were searched from 1 January 1994 to 19 January 2024. The search start date and end date were chosen based on the first and last paper found with the search terms. Papers were retrieved from two categories of interest: (1) 3D Printing AND (2) Temporal Bone models for surgical simulation. For each of these categories, Medical Subject Headings (MeSH) were used to ensure that the retrieval process was thorough. The search terms for these categories were (1) 3D Printing: “3D Printing” OR “3D-Printing” OR “Three-Dimensional Printing” OR “Three Dimensional Printing” OR “Stereolithography” and (2) Temporal Bone: “Temporal Bone”.

### Selection criteria

The selection criteria were that of articles that (1) were written in English, and which fulfilled all of the following requirements: (2) studied 3D printed adult temporal bone models used for surgical simulation or training, (3) contained a qualitative or quantitative comparison of 3D printed models with cadaveric temporal bones.

Articles were excluded if they (1) did not meet the inclusion criteria, (2) were review articles that did not contribute any original data, (3) did not develop 3D printed temporal bone models (4) did not contain or discuss results from a surgical simulation as defined as performing otological procedures on the models by ENT surgeons.

Two authors independently reviewed each study and reached a consensus on the studies to be included in the review.

**Fig. 1:**
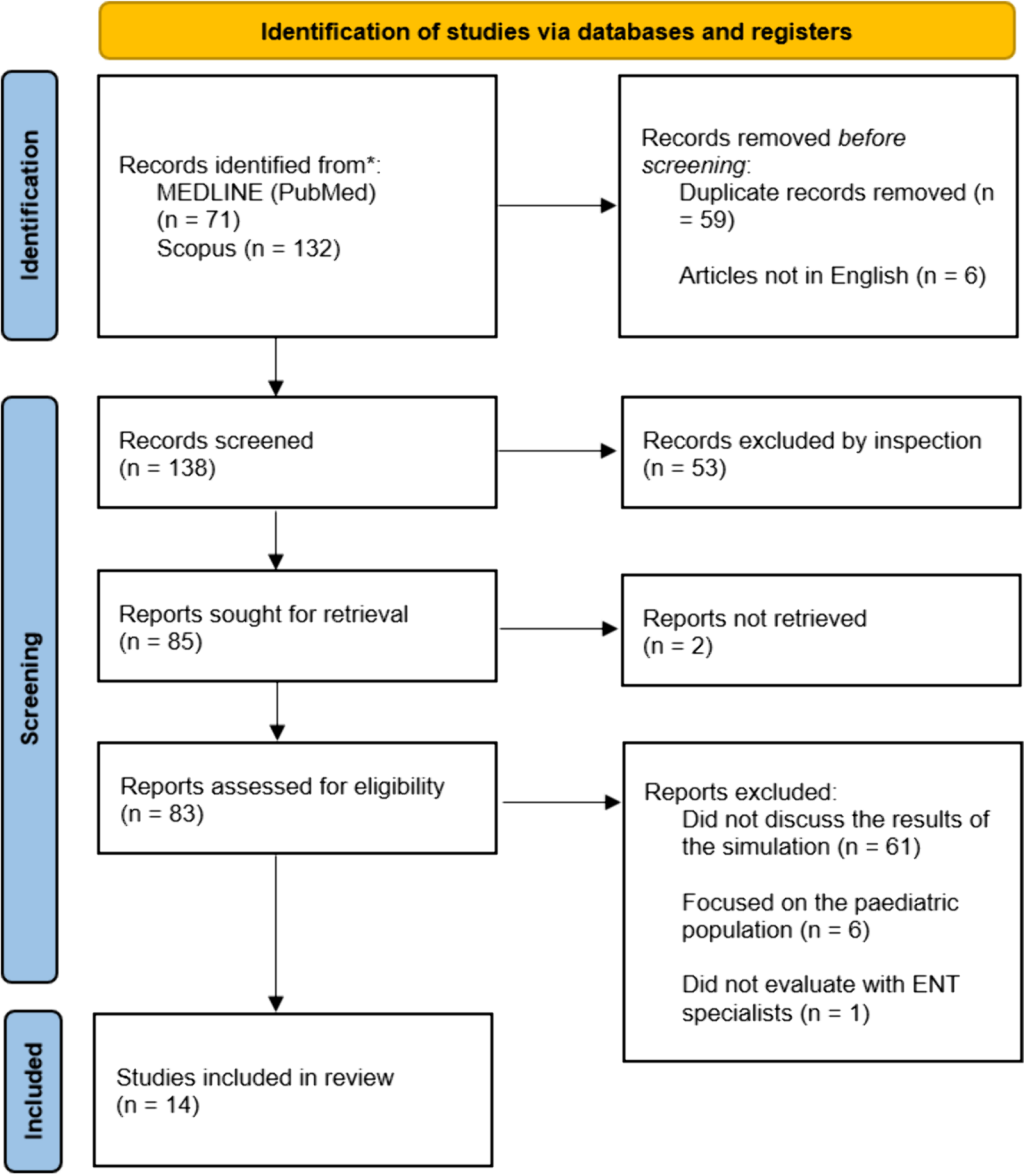
PRISMA flowchart for study selection

The literature search yielded 71 articles from PUBMED and 132 articles from SCOPUS. There were 59 duplicates and 6 non-english studies. Of these, 53 articles were excluded after reviewing their title and abstracts and 2 studies could not be retrieved. The remaining 83 papers were assessed against the selection criteria. From these, 68 articles were excluded for the following reasons: (1) did not use 3D printed temporal bone models (*n* = 30), (2) did not perform surgical procedures with ENT surgeons (*n* = 24), (3) review papers without original data (*n* = 6), (4) did not study adult temporal bones (*n* = 6). A total of 17 articles were included (see Fig. 1). Table S1 summarises the key findings of the articles included. The key findings of the literature review are that the ossicular chain (*n* = 7), mastoid air cells (*n* = 4), course of the facial nerve (*n* = 4), semi-circular canals (*n* = 2), chorda tympani (*n* = 1) and the cochlear labyrinth (*n* = 1) were not well-represented. This in turn affects the fidelity of the 3D printed temporal bone models to adequately simulate otological procedures.

## Methods

**Fig. 2:**
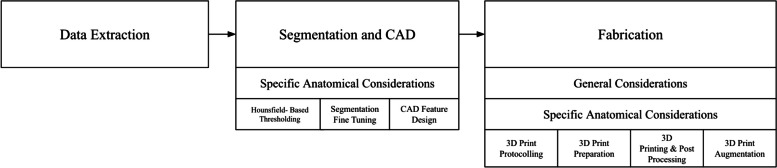
Outline of developmental phases and workflow of the temporal bone model

Based on the review the development phases of the 3D-printed temporal bone model were derived and described in a workflow (Fig. 2). An overview of the design considerations as well as the strategy and methods employed to address them was developed (Table 1).

**Table 1:** Overview of methods employed to address considerations for temporal bone model design and fabrication

Anatomical Region/ Feature	Design & Fabrication considerations	Strategy and methods employed (at segmentation, DfAM, post processing stages).
**General Considerations**		
**Model Material and Colour**	To have the model safe for drilling by high-speed surgical drills that produce fine particles.	Construct the model with material safe for high-speed drilling that produces fine particles.
	To have the model mimic the normal colour of the bone, reducing glare from Operating Theatre lighting.	Dye resin to a colour tint which minimises glare.
**Considerations at Specific Anatomical Regions**		
**Mastoid air cells**	To achieve differential demarcation of the Koerner Septum relative to the adjacent mastoid air cells.	Use fine detail slice-by-slice segmentation methods in addition to HU threshold method segmentation of key areas of the mastoid air cells.
	To achieve appropriate density of mastoid air cells.	
	To prevent print failures due to “cupping” i.e., formation of pools of uncured resin which prevent successful printing.	Orientate the model in a manner to prevent “cupping”.
**Tympanic Cavity Ossicular chain**	To model small sized and thin anatomical structures e.g., ossicles and tympanic membrane.	Use multiplanar reconstructions to suitably orientate the ossicular chain to allow for easier fine detailed segmentation.
	To mitigate impact of supports obstructing the key anatomy of the tympanic cavity.	Use manual support placement techniques to minimise support density and touchpoint size at key anatomical structures of the ossicular chain and along surgical corridors in the tympanic cavity.
	To model the course of the tympanic segment of the Chorda Tympani nerve which is not visible on CT.	Model the expected course of the Chorda Tympani nerve based on known anatomical landmarks.
	To model the appearance of the skull base boundary structures during otological surgery i.e., the dura of the middle cranial fossa, the transverse and sigmoid sinuses.	Use silicone to model the tympanic membrane, dura and dural venous sinuses.
**Facial nerve**	To maintain the demarcation and integrity of the facial nerve canal from the adjacent bone.	Use CAD Modelling techniques to demarcate the walls of the facial nerve canal as well as place resin drainage holes that allow flushing through it using millifluidic printing techniques.
	To prevent uncured resin from depositing inside instead of draining through the thin facial nerve canal.	Inject coloured silicone through the facial nerve canal to differentiate it from normal bone.
**Cochlea**	To model the detailed anatomy of the cochlea in order to facilitate cochlear nerve implant simulation.	Use detailed fine slice-by-slice segmentation methods to delineate the turns of the cochlear as well as the modiolus.
	To simulate the round window niche as a surgical landmark.	Use CAD modelling techniques to place demarcating features at these niches.
**Semicircular canals**	To prevent uncured resin from depositing inside the small channels instead of draining through them.	Use CAD modelling techniques to enlarge as well as place resin drainage holes within the channels which allow flushing through them using milli-fluidic printing techniques.
		Consider the orientation of the semicircular canals during model orientation.

This workflow through its phases and steps as exercised upon a case example is described.

### Phase 1: data extraction

An anonymised normal CT scan of the left temporal bone of a middle-aged female with slice thickness 0.6 mm, 120 keV and 137 mAs was utilised as the dataset. The Institutional Review Board waived ethics approval for this study (reference number 201806-00051).

### Phase 2: segmentation and CAD

The digital imaging and communication (DICOM) images were imported into an open-source image visualisation and segmentation software for processing (*3D Slicer* [5]). An initial segmentation of the bony anatomy of the temporal bone was performed using a Hounsfield Unit (HU) thresholding technique (range 415–2929 HU). Subsequently, fine-tuning of the segmentation as well as the addition of CAD features were performed using manual segmentation manipulations upon the relevant anatomy based on specific anatomical considerations detailed below.

### Mastoid air cells

**Fig. 3:**
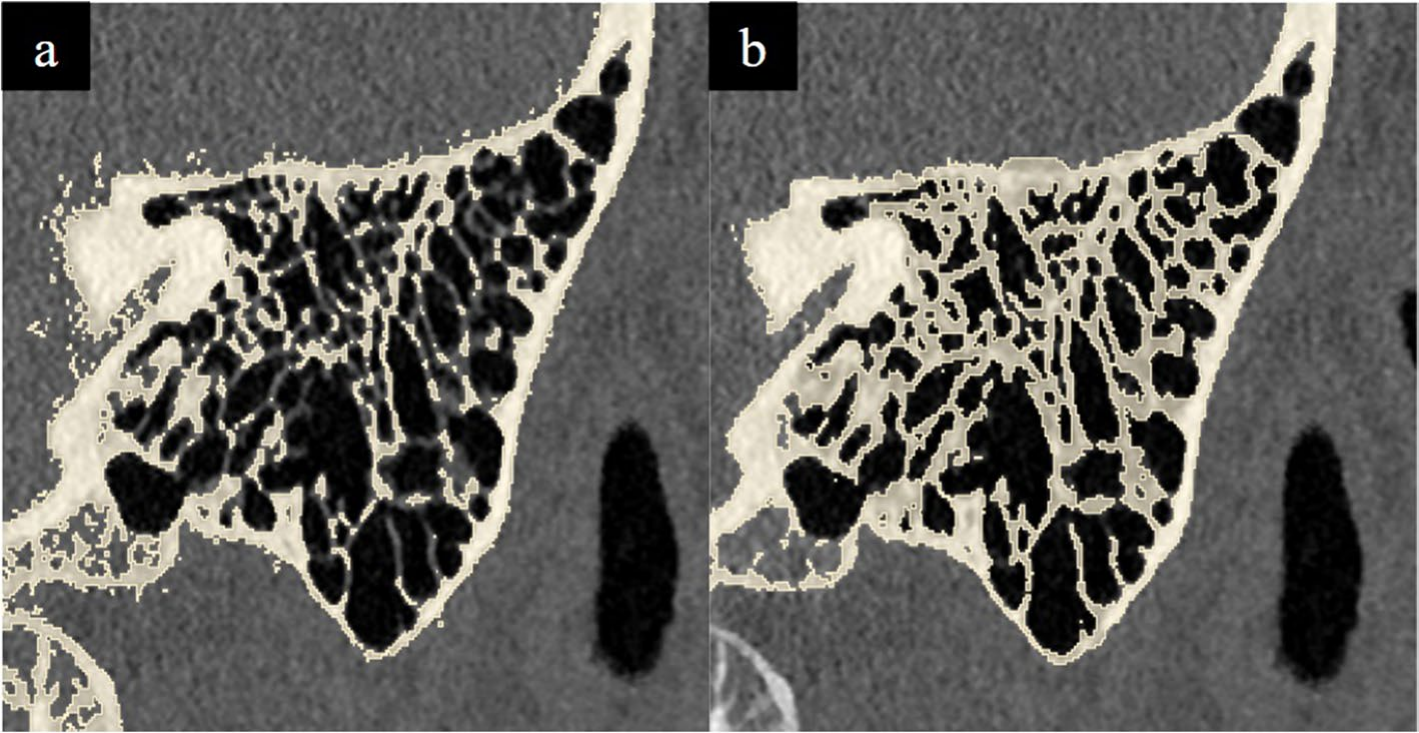
**a** HU thresholding results in incomplete segmentation of the mastoid septa, (i.e. appear grey and not yellow) due to their variable density. **b** Segmentation of these septa is achieved by using manual segmentation fine tuning

The mastoid air cells should be represented accurately as the first step to most otological procedures involve cortical mastoidectomy. The design and fabrication considerations in their modelling include achieving (a) differential demarcation of the Koerner’s septum relative to the adjacent mastoid air cells to facilitate recognition of entry into the middle ear cavity (b) an appropriate density of air-filled mastoid air cells. Techniques used included HU thresholding and segmentation fine-tuning to maintain anatomical accuracy while minimising the need for artifactual support structures to the self-supporting ‘honeycomb’ pattern of the septa (Fig. 3).

### Tympanic cavity

For the tympanic cavity, the objectives were to simulate procedures like stapedotomy and ossicular chain reconstruction. This required (a) detailed modelling and recreation of the thin tympanic membrane, (b) avoiding obstruction of key anatomy in the tympanic cavity by 3D printed supports, (c) accurate depiction of the ossicular chain despite its small size, (d) modelling of the stapes footplate and the chorda tympani nerve even with poor visualisation on CT, and (e) anatomically representing skull base boundary structures like the dura and dural venous sinuses which impart a different colour tinge as they are approached surgically.

**Fig. 4:**
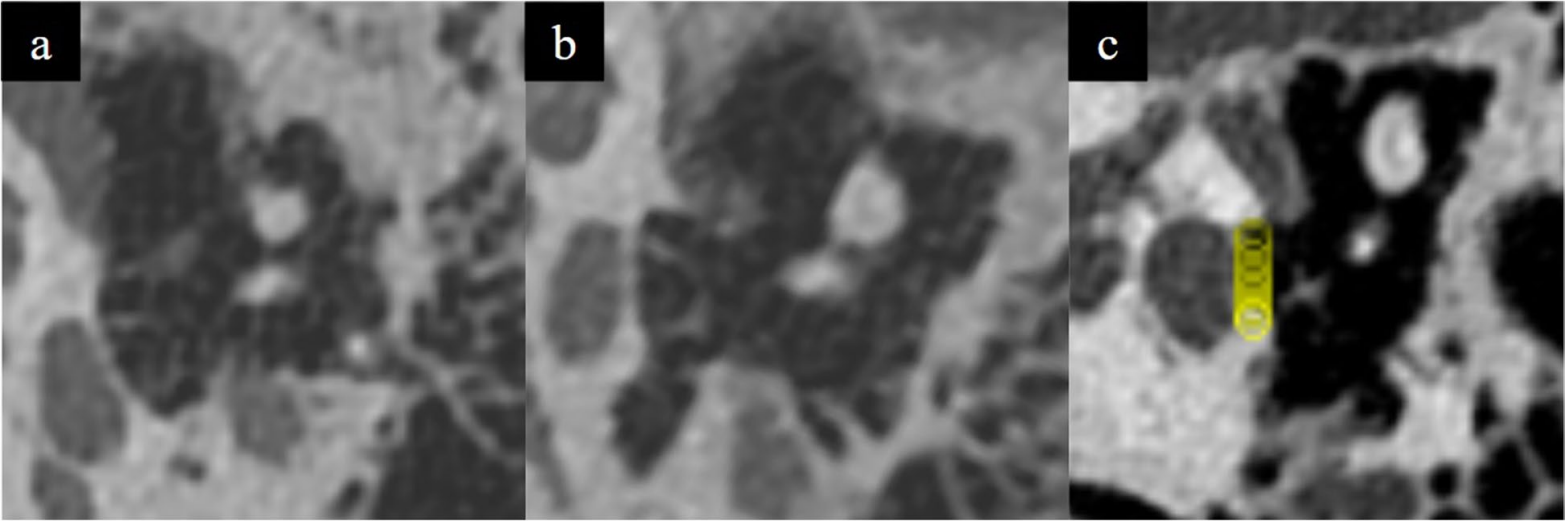
**a** Axial image and (**b**) multiplanar reconstruction of the temporal bone. **c** Note that multiplanar reconstructions orientate structures for easier segmentation e.g., the stapes footplate

**Fig. 5:**
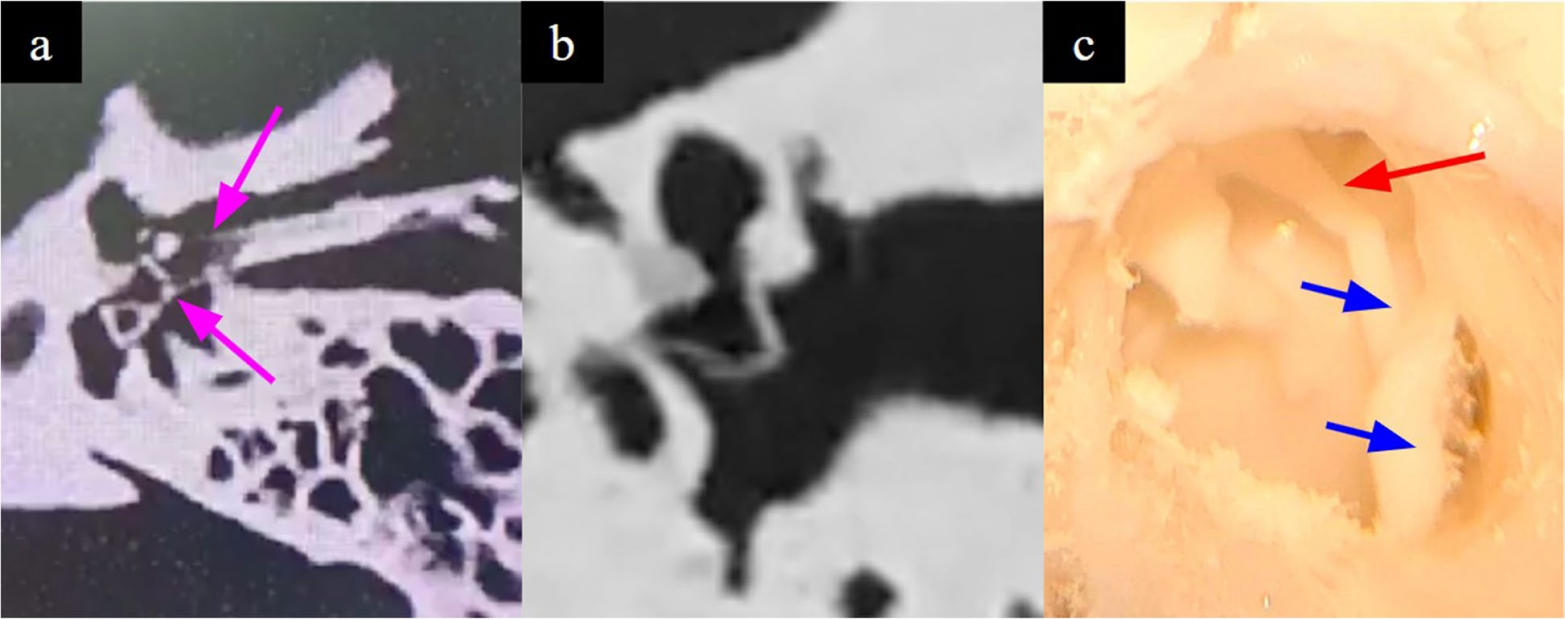
**a** Automatically generated support structures owing to the inverted vat polymerisation printing method in the tympanic cavity in this case traversing the external acoustic meatus and attaching to the lentiform process of the incus and to the malleus (pink arrows). **b** These are minimised by manual techniques of support number and size generation. Images are from CT scan of the model. It is important for the user of the simulator to not mistake inverted vat polymerisation support structures from anatomical structures, in **c**, the manubrium of the malleus is indicated by a red arrow, while supports arising from its inferior aspect as well as in the tympanic cavity superficial to it are indicated by blue arrows

**Fig. 6:**
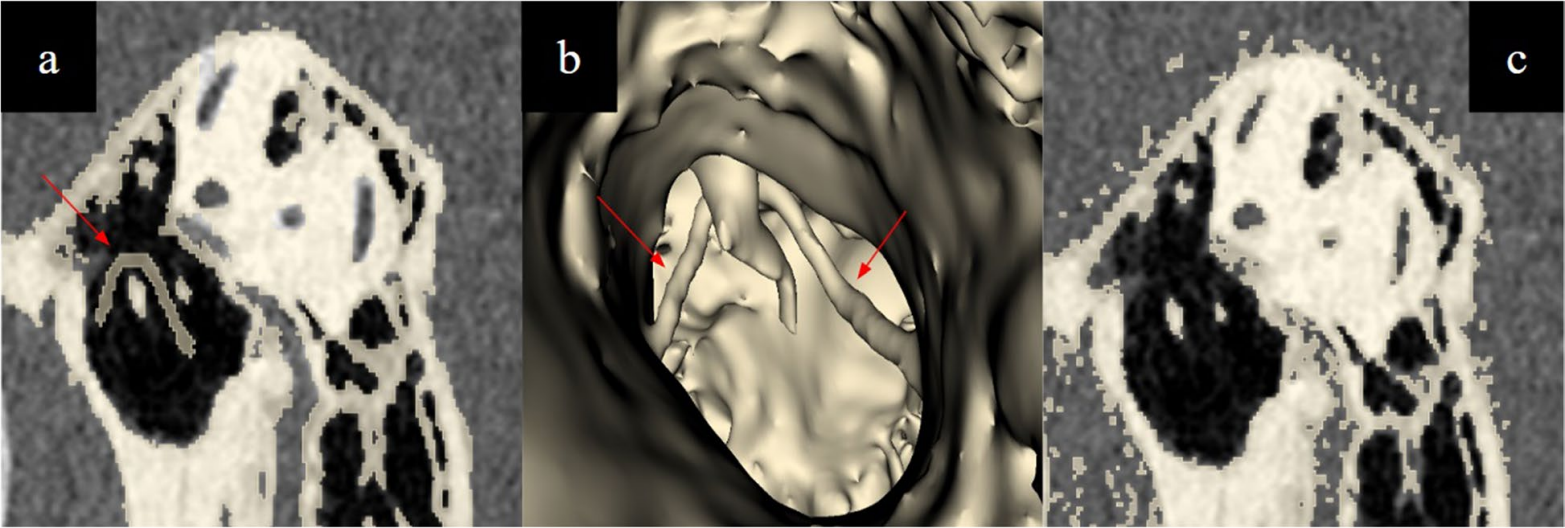
**a** CAD modelling and (**b**) 3D rendering of the expected course of the tympanic segment of the chorda tympani nerve (arrow) which courses medial to the handle of the malleus. **c** This is not normally visualised on CT due to its thin size and low density. The nerve is identified and preserved during ENT procedures involving the middle ear

**Fig. 7:**
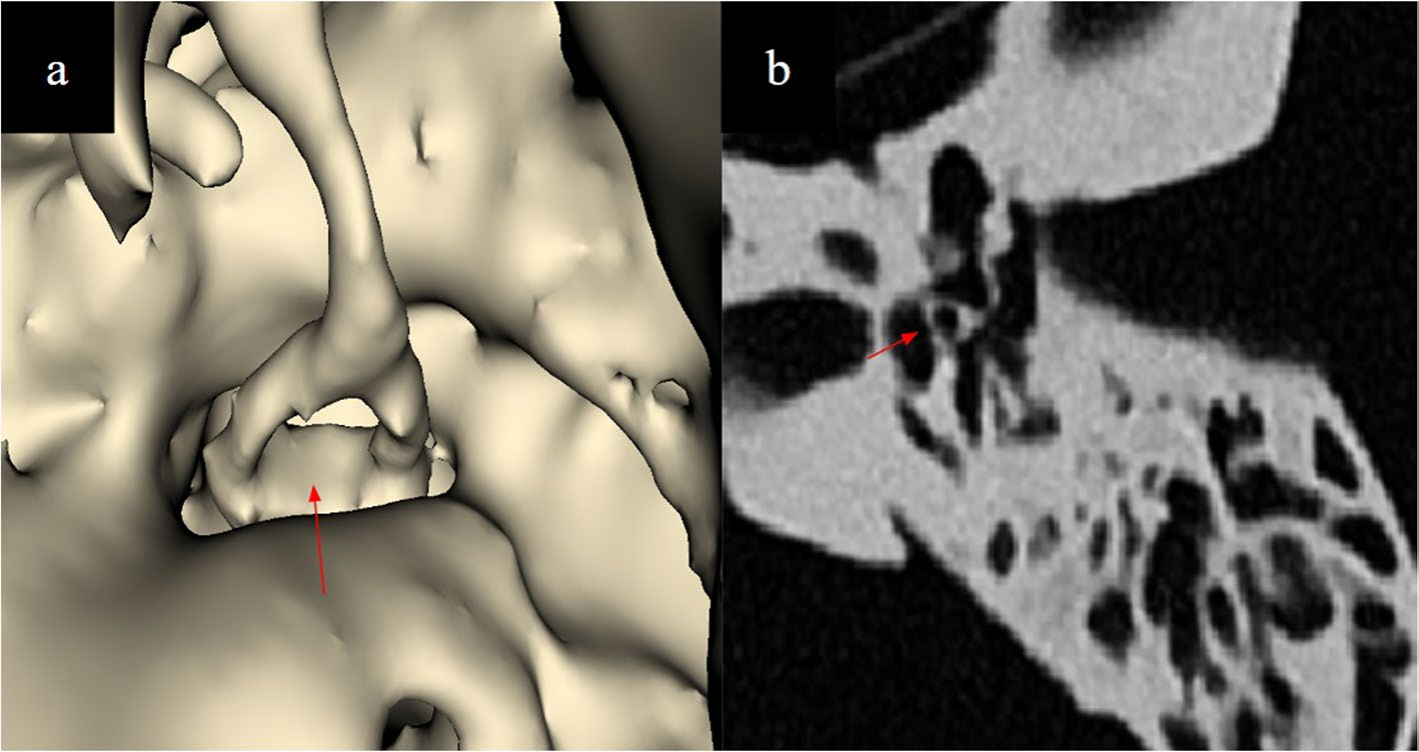
**a** CAD modelling of the stapes footplate located perpendicular to the opening of the oval window and both crus of the stapes (**b**) imaging correlate on CT scan of the model. This is not well visualised on CT despite HU thresholding due to its thin diameter and low density

Techniques employed included (a) 3D printing utilising inverted vat polymerisation techniques, (b) performing multiplanar reconstructions on the temporal bone to aid in segmentation (Fig. 4), and (c) utilisation of manual 3D printer support placement techniques to minimise amount of supports and support touchpoint size in the tympanic cavity (Fig. 5). CAD design techniques were used for modelling the tympanic segment of the chorda tympani nerve based on anatomical landmarks of its expected course (Fig. 6), as well as for modelling of the stapes footplate (Fig. 7). Silicone was also used to model the tympanic membrane and skull base structures as elaborated below (refer to section below - *Fabrication: 3D Print Augmentation*).

### Facial nerve

**Fig. 8:**
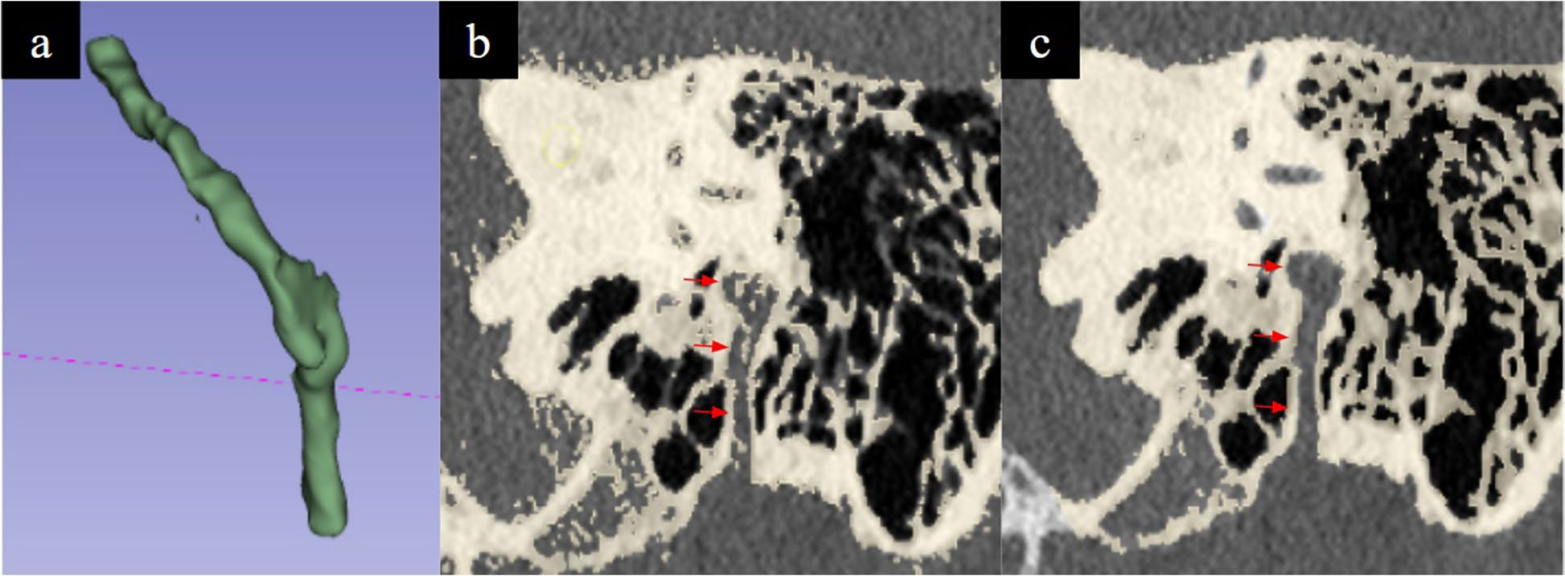
**a** CAD modelling of the facial nerve course (**b**) removes the artefacts seen within its lumen (red arrows) and (**c**) ensures its continuity to prevent leakage of silicone from it into the adjacent mastoid air cells or tympanic cavity

**Fig. 9:**
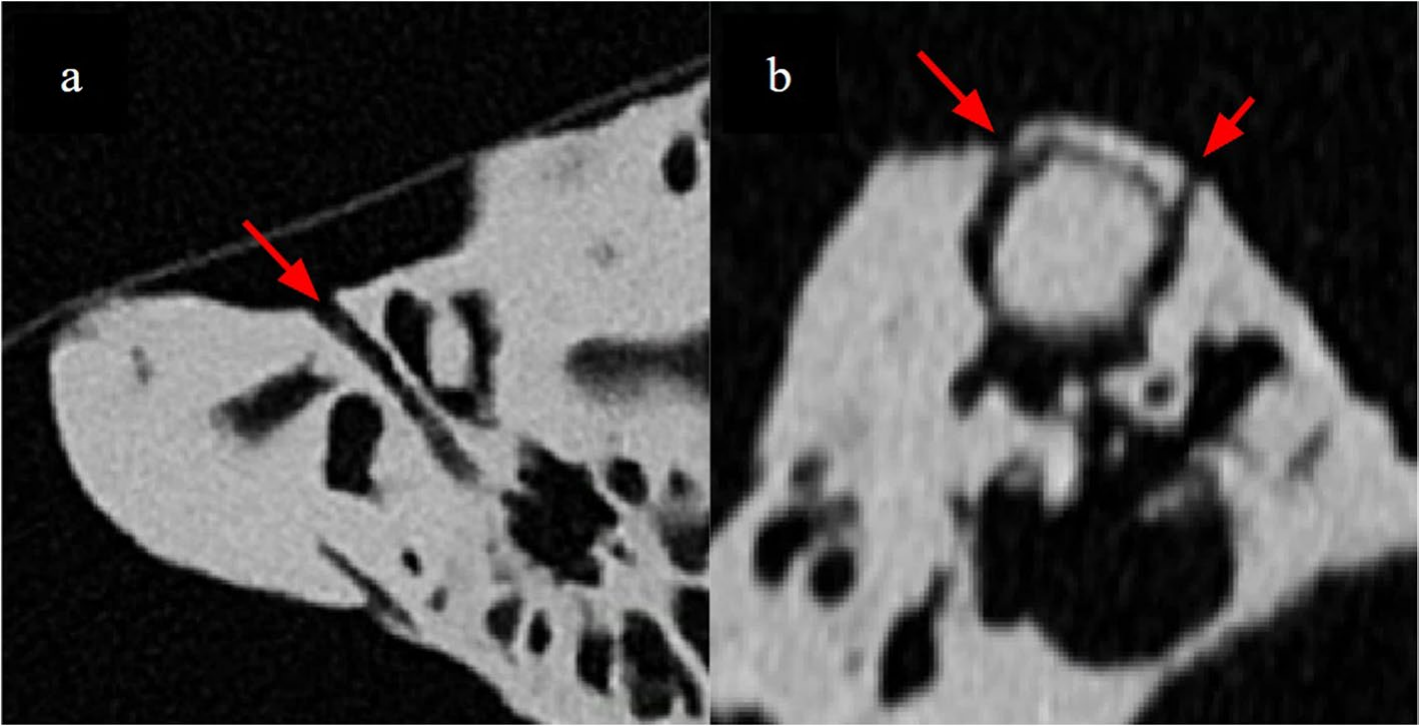
**a** Non-anatomical resin drainage channels (arrows) created by CAD modelling extending medially from the tympanic segment of the facial nerve, and (**b**) at the superior aspect of the superior semicircular canal, seen on multiplanar reconstructed CT imaging. These channels are placed in locations to allow millifluidic post processing (see step: 3D printing and post processing) while not interfering with surgically important anatomical landmarks

**Fig. 10:**
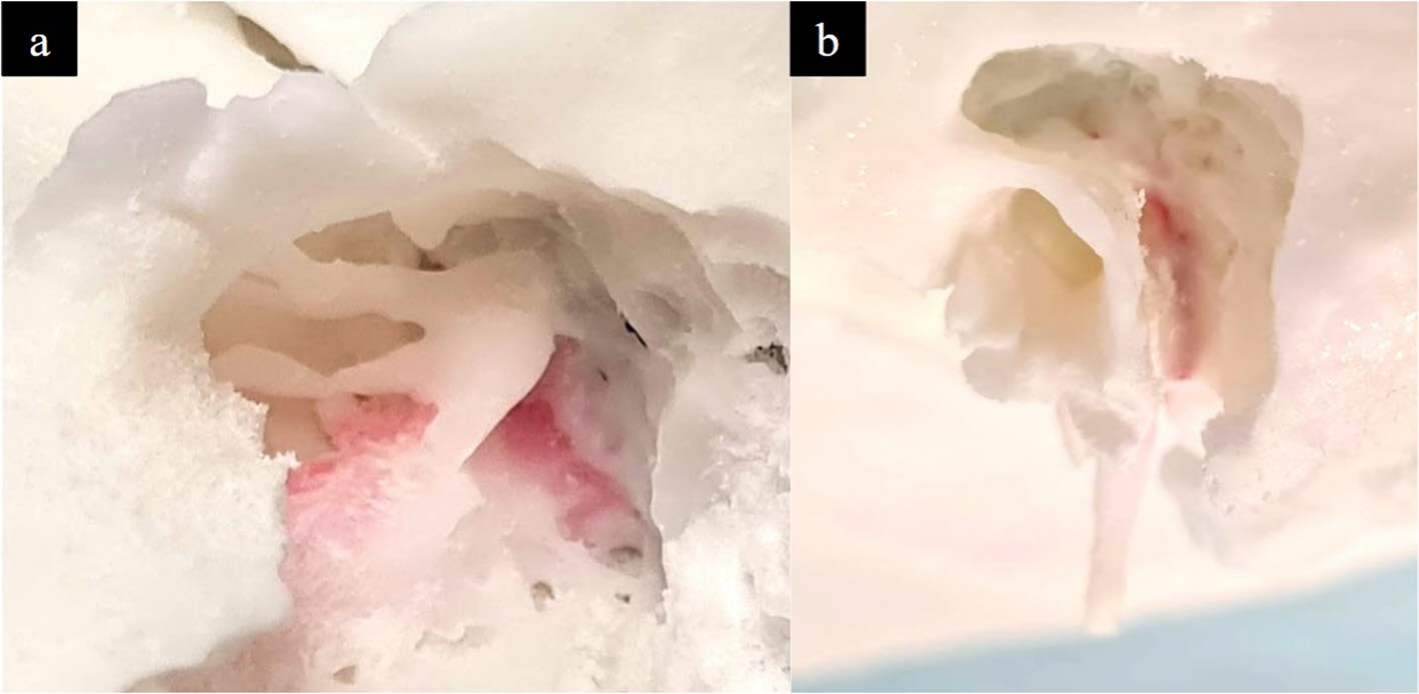
Presence of coloured silicone in the tympanic segment of the facial nerve canal to differentiate it from normal bone on two models. In (**a**) the ossicles are located superficial to the canal

The lateral semicircular canal, mastoid segment of the facial nerve and the intraosseous segment of the chorda tympani nerve branching from the facial nerve help demarcate the borders of the facial recess, a crucial part of cochlear implant surgery. Design and fabrication considerations include (a) demarcation of the facial nerve as a separate landmark in the setting of a single colour and material inverted vat polymerisation print as well as its potential dehiscence which is has a prevalence in up to 57% [6] in normal anatomy (b) prevention of uncured resin from depositing inside instead of draining through the thin facial nerve canal. Our solutions were to: (a) CAD model the facial nerve canal to ensure integrity of its walls and prevent silicone leakage (Fig. 8), (b) place resin drainage holes at selected segments of the facial nerve canal which allow flushing through it using millifluidic printing techniques (Fig. 9), (c) inject coloured silicone through the facial nerve canal via the stylomastoid foramen to differentiate it from normal bone (Fig. 10).

### Cochlea

**Fig. 11:**
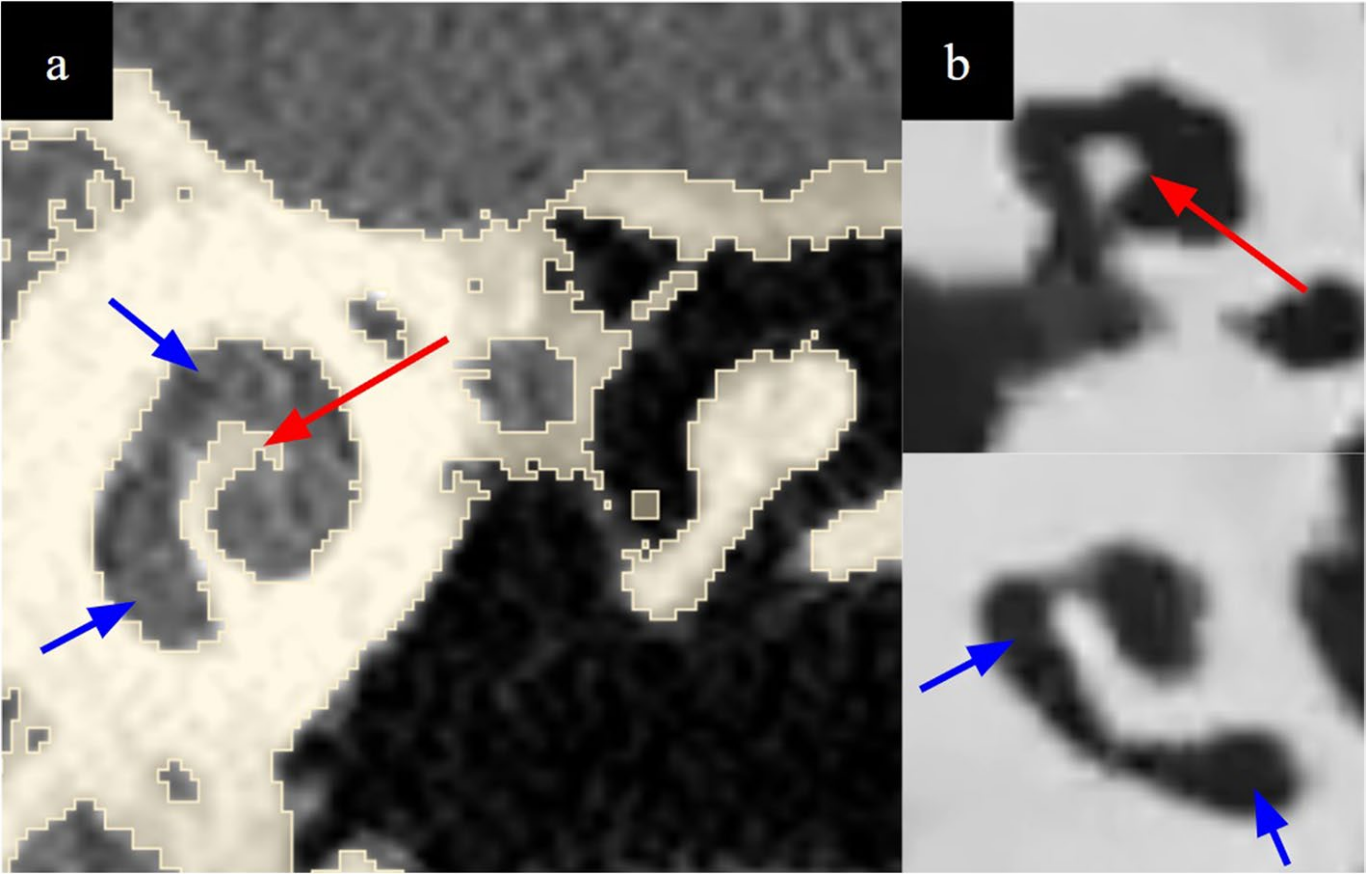
**a **The scala tympani (blue arrow) and modiolus (red arrow) on segmentation renderings and (**b**) concomitant CT imaging of the model

**Fig. 12:**
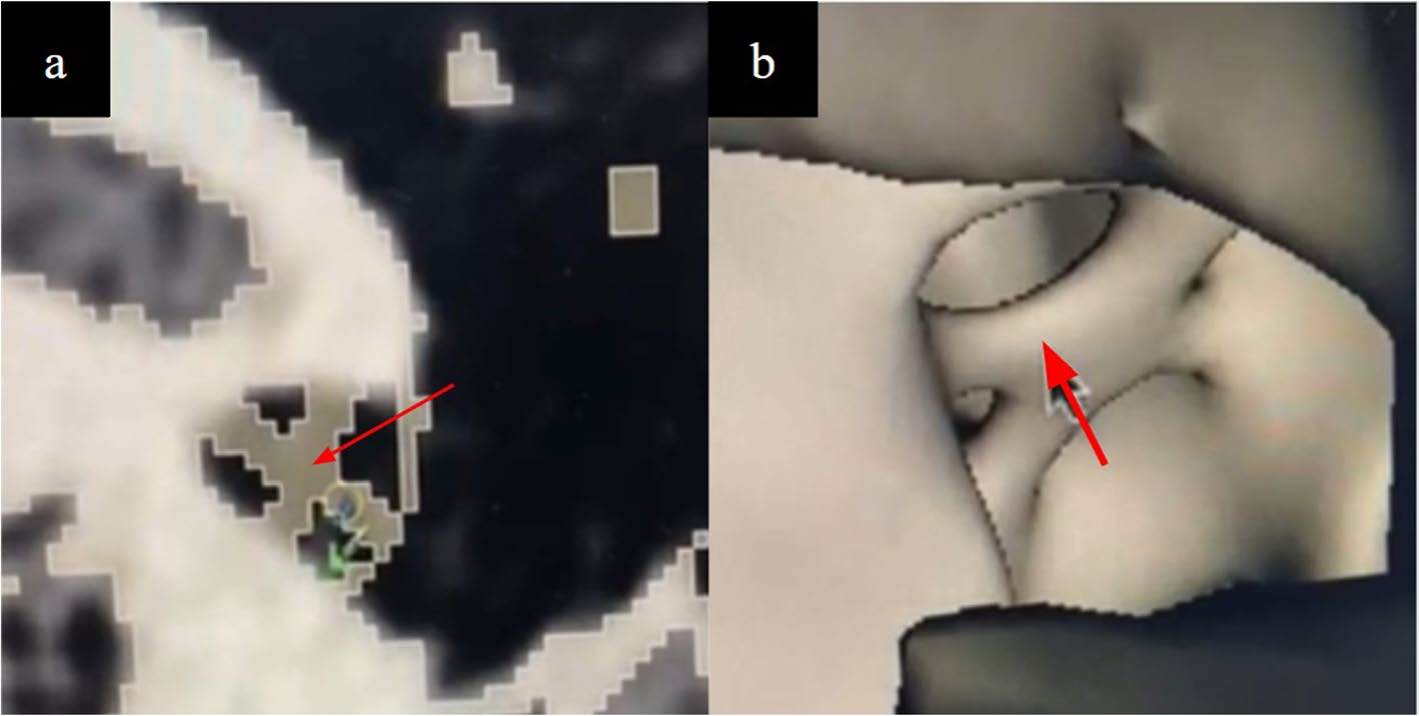
**a** CAD modelling and (**b**) 3D rendering of the round window membranous opening depicted with an X-shaped structure, which although non-anatomical serves to delineate its location perpendicular to the opening of the oval window and both crus of the stapes. This is not well visualised on CT due to its thin diameter and low density

Accurate modelling of the cochlea is necessary to simulate cochlear implant electrode insertion via a cochleostomy or the round window. The two required features were (a) a conical and spiral shaped canal representing the scala tympani winding for two-and-a-half turns around a central structure representing the modiolus (Fig. 11) (b) the round window niche. The following techniques were used, (a) segmentation fine tuning in the region of the scala tympani to ensure patency of this channel, (b) CAD modelling of the modiolus which may not be well visualised on CT, (c) CAD modelling of the round window membranous opening which was depicted as an X-shaped structure (Fig. 12).

### Semicircular canals

**Fig. 13:**
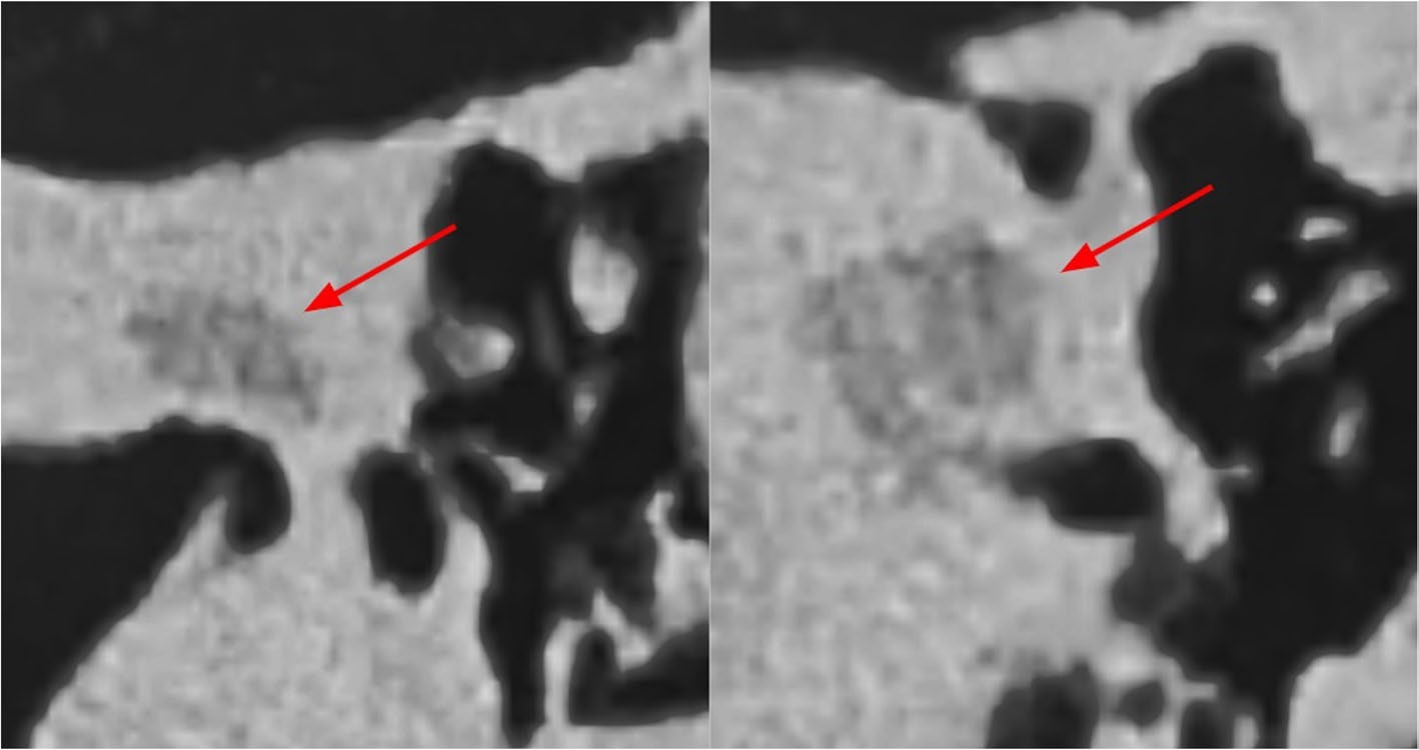
Presence of uncured resin appearing as material of density intermediate to cured resin and air within the cochlea (arrows)

Modelling of the semicircular canals is necessary to simulate labyrinthectomy and superior semicircular canal dehiscence surgery. We needed to prevent uncured resin during the inverted vat polymerisation printing process from depositing inside the narrow channels and occluding instead of draining through them (Fig. 13). This was achieved by (a) enlarging the lumen of the channels via CAD modelling (b) placement of two drainage channels extending from the superior semicircular canal to the middle cranial fossa via CAD modelling (Fig. 9). These together with fabrication techniques described in the subsequent section aided accurate modelling.

Following these segmentation and CAD manipulations, a separate CAD software (Autodesk Meshmixer, Autodesk Inc., CA, USA [7]) was used to optimize the model for 3D printing. The Sculpt tool was used with the ShrinkSmooth brush to smoothen the surface of the model in order to minimise the number of external supports. The ZipperEdge tool was used to close artifactual holes in the surface of the model which arose due to the segmentation process. The Plane Cut tool was used to isolate the area of interest so as to minimise the amount of material required to print the model and therefore the cost of the model.

### Phase 3: fabrication

The derived file was then prepared for fabrication in the steps of 3D print protocolling, print preparation, printing and print augmentation considering general and specific anatomical design and fabrication considerations. These are as detailed.

### Step 1: 3D print protocolling

Print protocolling refers to the process of printer setting optimization in the printing software (*Preform, Formlabs, MA, USA* [8]).

### Anatomical region-specific considerations: tympanic cavity, semicircular canals

Considered placement of the inverted vat polymerisation support structures on the model is important with an ideal inverted vat polymerisation model having a minimal number of internal supports and as small a support touchpoint size (i.e. the diameter of the support) as possible, especially in key regions such as the tympanic cavity. To achieve this while minimising the time taken for print protocolling, external supports of the model which supported its external surface were automatically generated while supports within the cavities of the model were placed manually (Fig. 5). During manual support placement, the minimal number of internal supports to adequately support the internal structures with the smallest possible touchpoint size (0.20 mm), were placed in a manner which could not be inadvertently mistaken for normal structures.

**Fig. 14:**
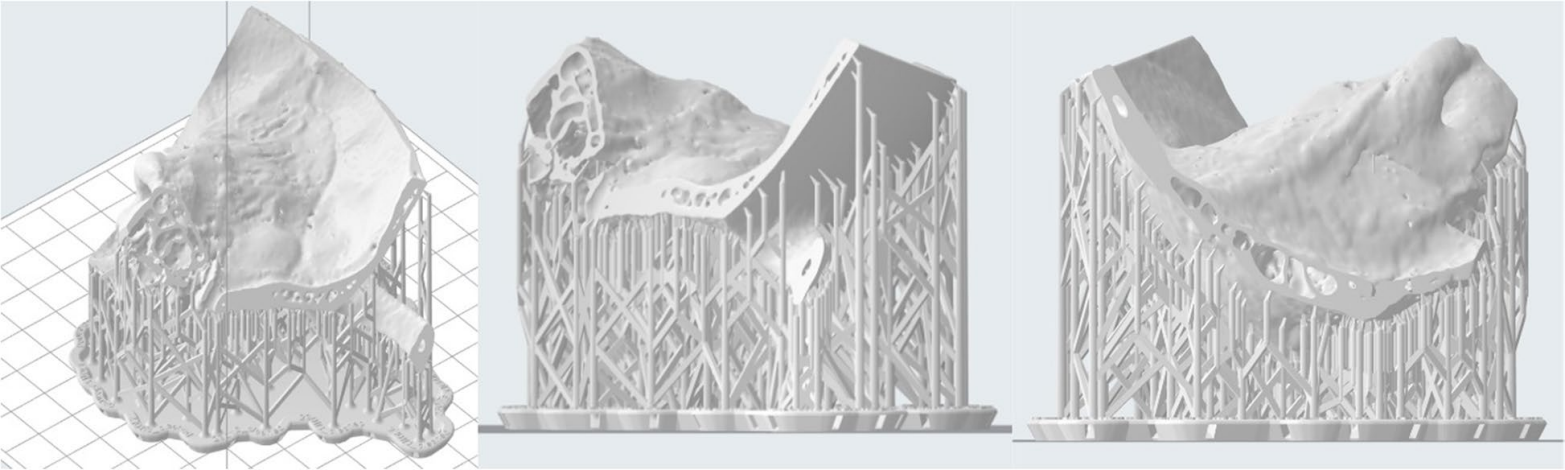
Orientation of the model on the print bed (various aspects shown)

Optimal orientation of the model on the print bed so as to allow for maximal drainage of resin from the semicircular canals with minimal residue of uncured resin was also important (Fig. 14). This was done considering that the print bed would move upwards from the resin tank, and also considering the position and orientation of the drainage holes extending from the superior semicircular canal. It was found that the optimum orientation of this model was with the Eustachian tube oriented downwards to the print bed, at an angle of approximately 90°.

The finest layer thickness achievable on this software for this resin (0.05 mm) was chosen to ensure the structures were printed in adequate detail.

### Step 2: 3D print preparation

Print preparation refers to the process of printer preparation to achieve the desired print qualities.

**Fig. 15:**
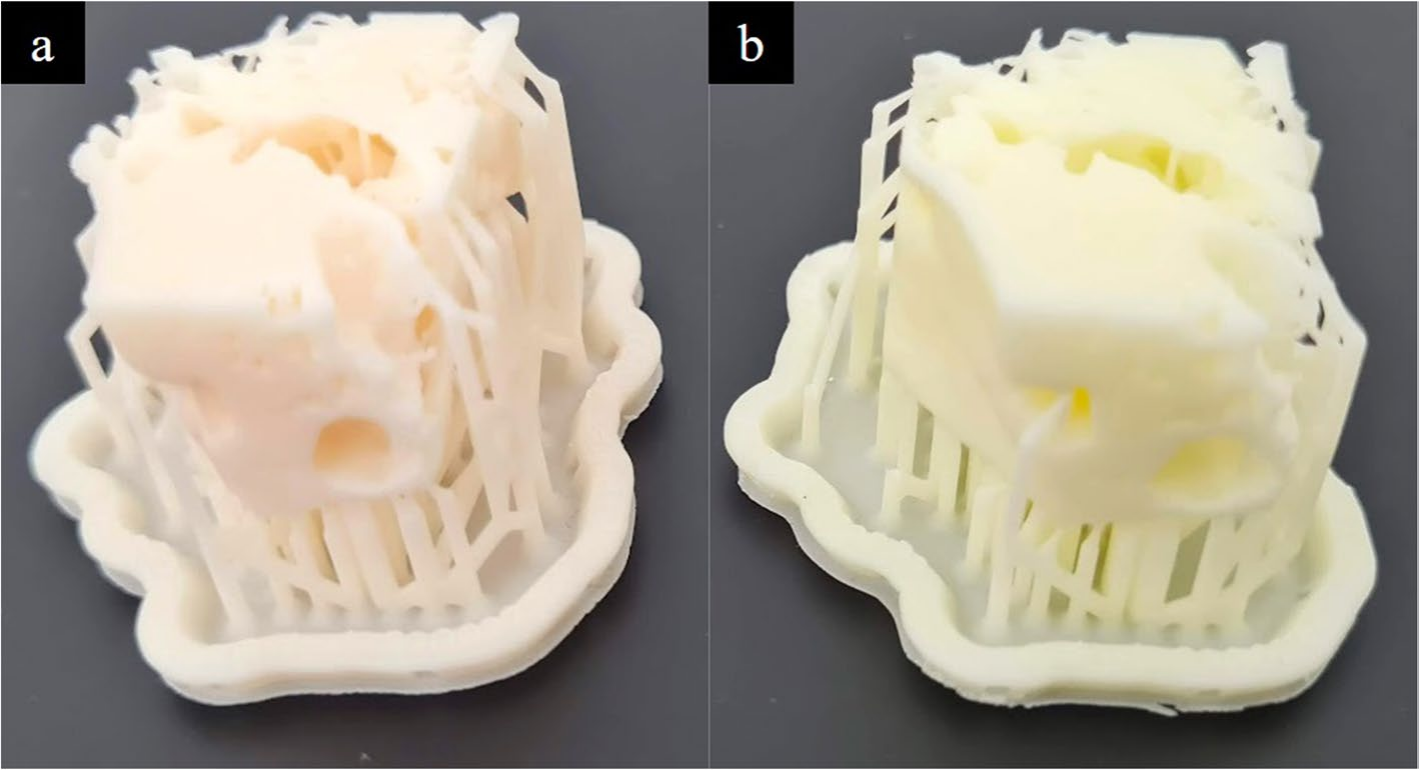
**a** A segment of the model printed with White Resin dyed with yellow and magenta pigment compared to (**b**) with undyed White Resin. The dyed resin was found to produce a similar appearance under the light of an operating microscope as that of physiological temporal bone

Of the available 3D printing techniques, inverted vat polymerisation was used as it produces accurate models that have haptic and anatomic characteristics suitable for temporal bone drill simulation [9]. Inverted vat polymerisation resins are relatively economical [10] and the resin we chose (White Resin from Formlabs Inc) was used based on the studies of its safety characteristics and as it was able to be dyed using the Formlabs Color Kit) fulfils Occupational Safety and Health Administration (OSHA) actionable levels and Permissible Exposure Limits during drilling [11]. We achieved a colour similar to bone by adding yellow pigment to White Resin in a 1:500 ratio, with Magenta pigment subsequently added to this mixture in a 1:2000 ratio and this helped prevent excessive glare when examined with an operating microscope (Fig. 15).

### Step 3: 3D printing and post processing

This step refers to the process of printing as well as post processing of the print i.e., washing and curing. The print time was 6.75 h. The 3D-printed model cost US $9.88 to print.

After printing and general washing of the model with Isopropyl Alcohol (IPA) as per the manufacturer’s recommendation, special care was taken to post-process specifically the small calibre facial nerve canal, as well as the drainage channels from the superior semicircular canal that were created by CAD modelling. This was performed using a protocol recommended for millifluidic geometry printing with inverted vat polymerisation, utilising repeat cycles of syringing of IPA, washing and drying of these channels [12].

### Step 4: 3D print augmentation

This step refers to the process of adding features to the model to improve the fidelity of its key anatomical regions to normal physiology. To achieve this, coloured silicone was applied on the model to (1) simulate the skin and soft tissue layer and the tympanic membrane, (2) delineate the skull base and the transverse and sigmoid sinuses, (3) simulate the facial nerve. Although this process was simple and only took approximately 15 min, it greatly enhanced the anatomical likeness of the model.

### Step 4.1: tympanic cavity

**Fig. 16:**
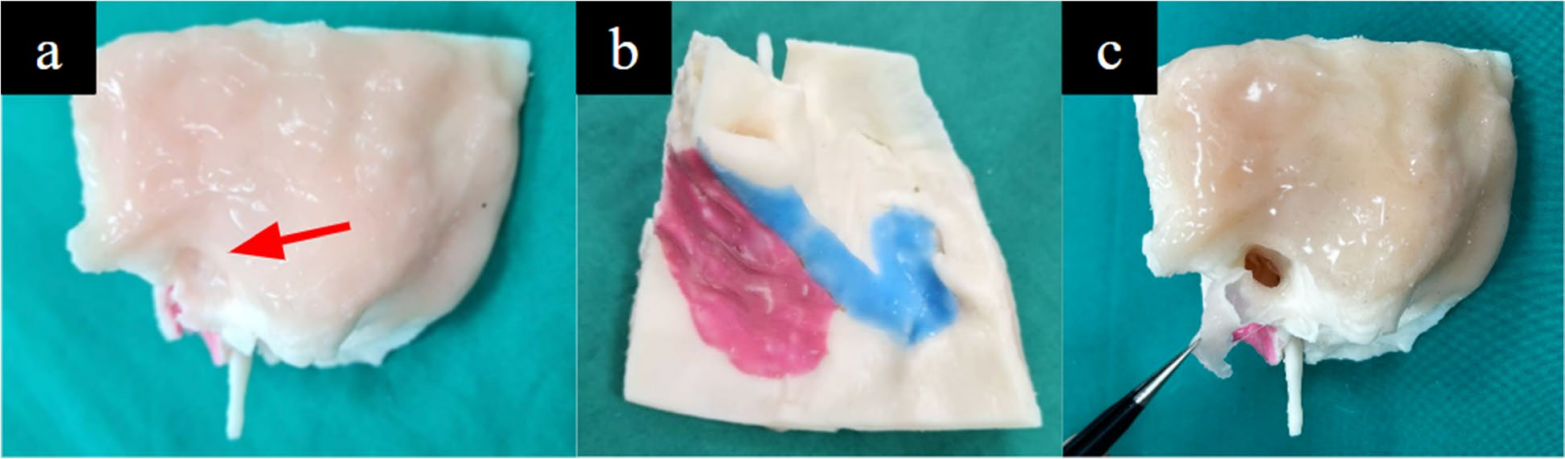
Silicone applied upon the model including (**a**) the tympanic membrane intact (arrow), (**b**) tympanic membrane retracted with forceps and (**c**) the middle temporal fossa dura (red) and dural venous sinuses namely the transverse sinus - sigmoid sinus junction (blue)

Silicone (Smooth-On Ecoflex 00–30) was dyed with Smooth-On Silc Pig colour pigment in suitable colours that matched these structures and applied onto the model (Fig. 16).

### Step 4.2: facial nerve canal

**Fig. 17:**
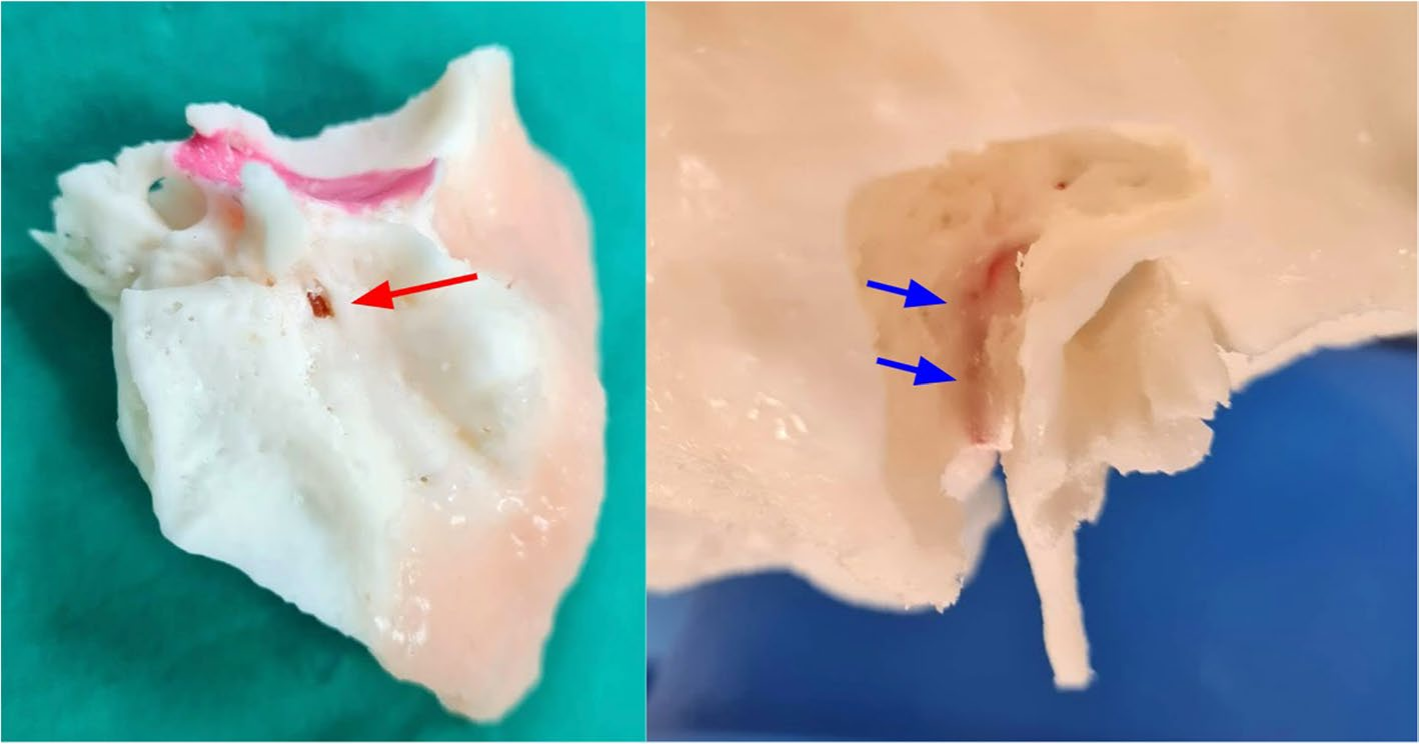
Silicone applied through the stylomastoid foramen (red arrow) to demarcate the course of the facial nerve (blue arrows)

Silicone (Smooth-On Ecoflex 00–30) was dyed with Smooth-On Silc Pig colour pigment and introduced with a small 22G needle through the stylomastoid foramen to dye the course of the facial nerve in its mastoid and tympanic segments (Fig. 17).

A simulation model was then created via a process of iterative design and assessed in stages by nine otolaryngology residents and attendings with an average of 8.1 years of training (range 1–13 years). Changes to the model were made based on their feedback and the latest version of the model was assessed by a senior otolaryngologist with 15 years’ experience. The final model was evaluated with a two-step validation process. Firstly, CT scan images of the temporal bone model were obtained. These images were then assessed for the internal structure of the model, following which measurements from the images were compared with the original scans to compare the dimensional accuracy of the model. Secondly, multiple otological procedures were performed on the model by the senior otolaryngologist to evaluate the haptic feedback of the model. Comments were collected on the (1) General appearance and (2) Anatomic likeness during tympanomeatal flap elevation, mastoidectomy, facial recess dissection, stapedotomy, round window dissection and cochlear implantation using a cochleostomy approach.

## Results

The model was dimensionally accurate to the original scan with sub-millimetre accuracy. Measurements taken of the external auditory canal at the scutum were 9.13 mm for the temporal bone model and 8.91 mm from the original CT data. For the internal acoustic meatus, measurements were taken at the porous acousticus and were found to be 5.04 mm for the temporal bone model and 4.90 mm for the original.

**Fig. 18:**
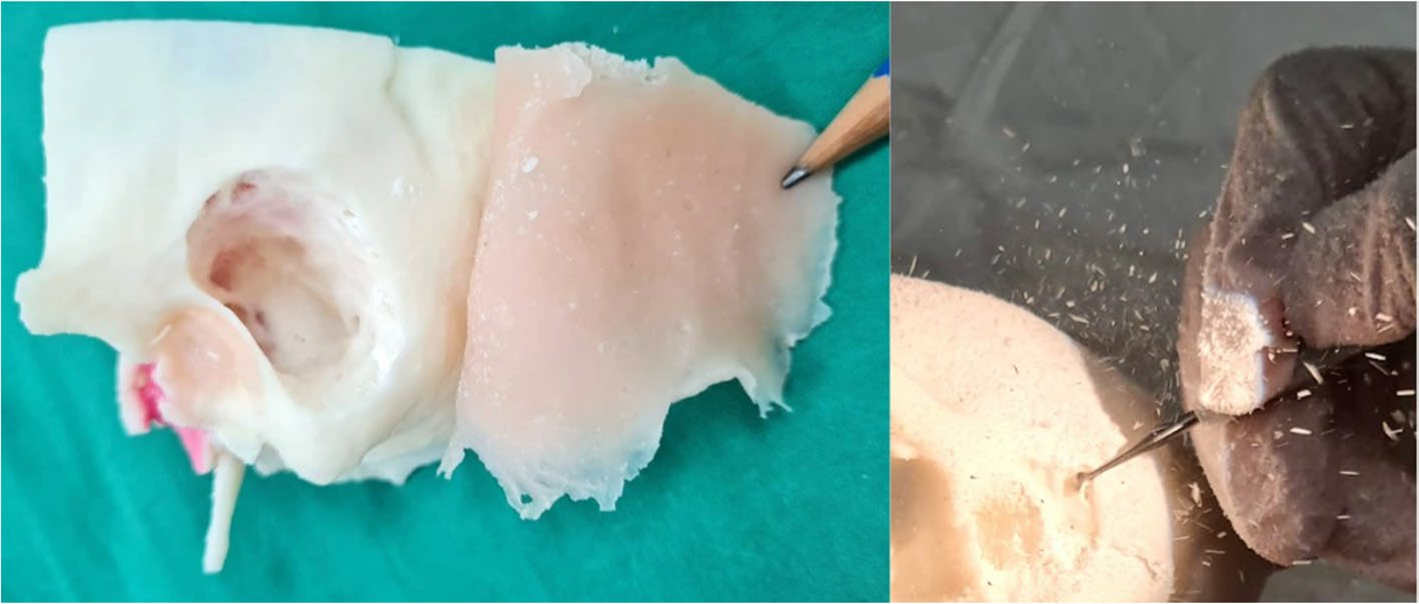
Simulation of Mastoidectomy. The silicone ‘skin flap’ is reflected posteriorly. Note presence of airborne particle dispersal on high-speed burr drilling

**Fig. 19:**
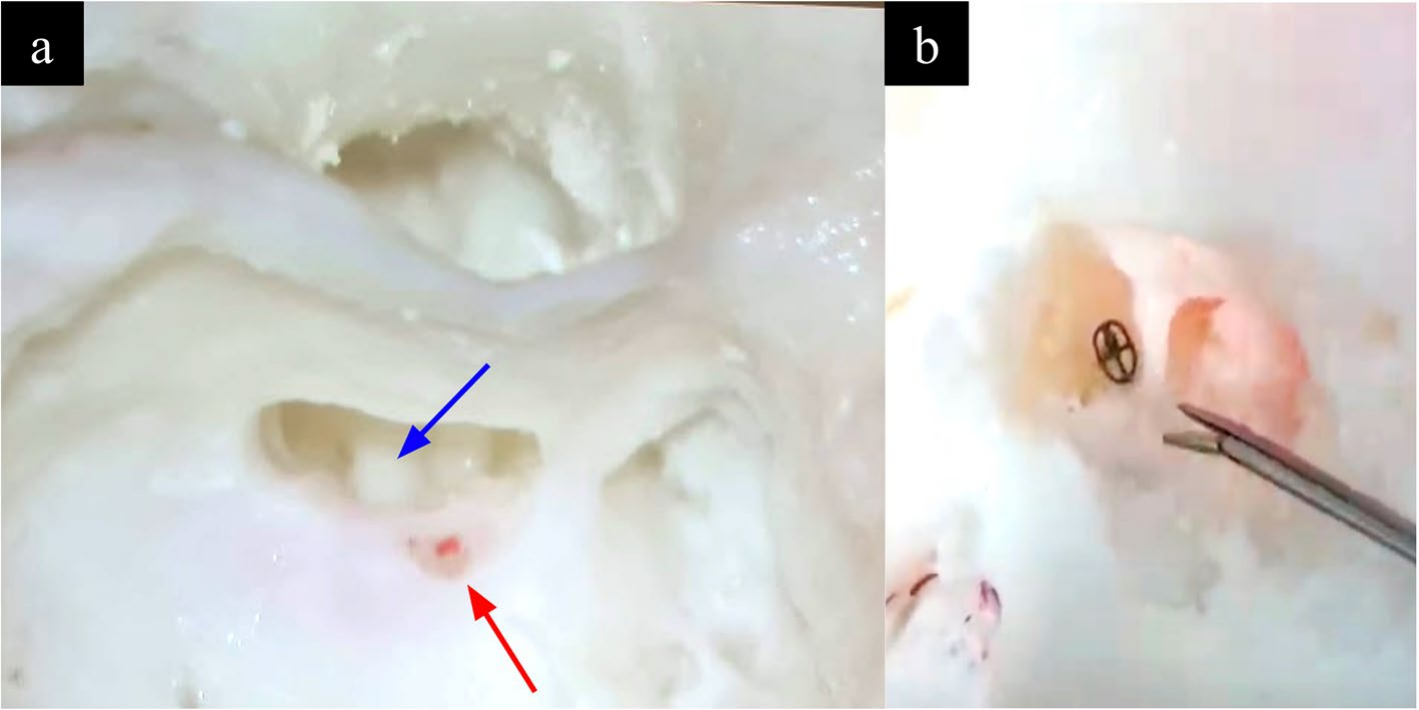
**a** Simulation of facial recess dissection, revealing the ossicles (blue arrow) and tympanic segment of the facial nerve canal with a red hue demarcating posterior extent of dissection (red arrow). **b **Simulation of ossiculoplasty with a TORP (total ossicular chain reconstruction prosthesis)

**Fig. 20:**
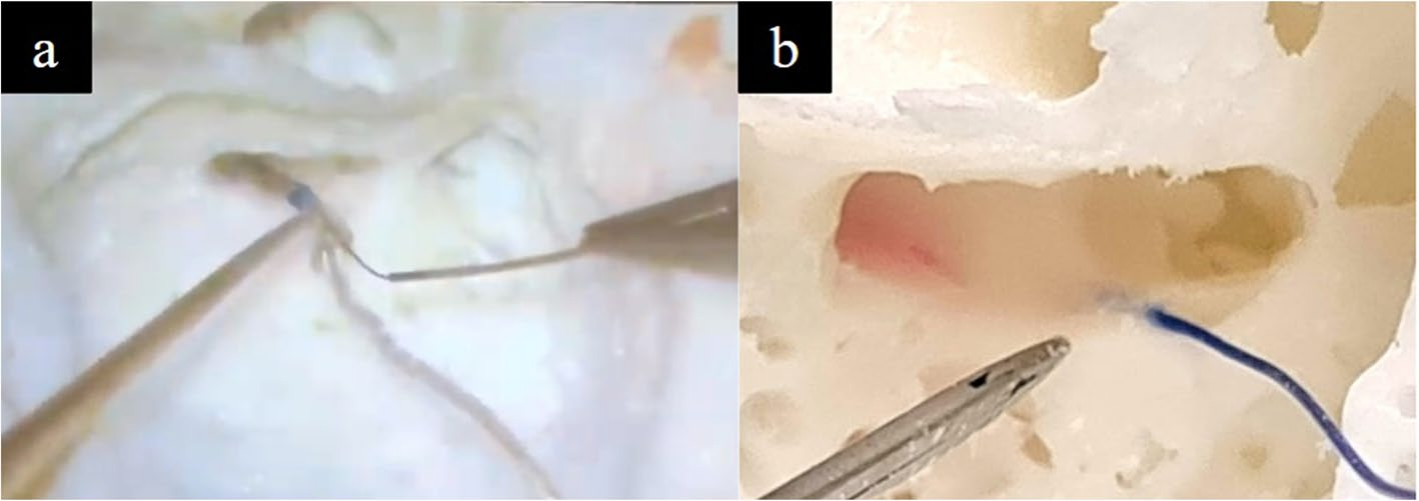
Simulation of CI surgery via cochleostomy with (**a**) an actual CI and (**b**) a suture to simulate the stimulator wire, the facial nerve canal is seen in red

Assessment by the senior otologist found that the model has satisfactory detail, colour and performance on high-speed drilling as compared to normal temporal bones. Multiple otological surgical procedures done on the model mostly found to be satisfactory (See Figs. 18, 19 and 20). Table 2 consolidates the key findings of the assessment.

**Table 2:** Assessment of general features and performance of the latest model version in simulating various surgical procedures

Domain	Assessment
General features of model	The model has generally satisfactory detail, colour and performance on high-speed drilling compared to normal temporal bone in most of its key regions of anatomy, including the mastoid air cells, tympanic cavity, facial nerve canal, cochlear, semicircular canals.
	Limitations of the model include fixation of the ossicular chain, presence of support struts in the tympanic cavity, absence of depiction of the intra-osseous course of the chorda tympani nerve.
**Surgical procedure performed on model**	
Tympanomeatal flap elevation	Satisfactory
Mastoidectomy	Satisfactory
Facial recess dissection	Mostly satisfactory, the intra-osseous course of the chorda tympani nerve was not depicted on the model.
Round window dissection	Satisfactory. It was necessary to recognise the structure demarcating the round window as non-anatomical.
Stapedotomy	Mostly satisfactory, the ossicular chain was not mobile but the oval window was able to be accessed and a simulated piston placed. It was necessary to recognise support structures as non-anatomical.
Cochlear implant (CI) surgery via cochleostomy	Satisfactory

## Discussion

This study reviewed the prior literature on the features and development methods. It described a development workflow for 3D-printed temporal bones. It also demonstrated the feasibility of developing 3D-printed temporal bones for complex otological surgery of the middle and inner ear. The temporal bone model that was developed was cost-effective at USD$6.09, it was also developed using resin which is found to be safe for simulating procedures. From the results, we demonstrate a sub-millimetre accuracy of the temporal bone model with respect to the original scan data. As for regulatory considerations, the segmentation software, 3D Slicer, is currently not approved by the FDA. However, the process illustrated in this article can easily be replicated with other segmentation software.

Limitations of this study include (a) the fixation of the ossicular chain limiting the performance of the model on stapedotomy and in simulating ossicular chain surgical procedures, (b) the absence of depiction of the intra-osseous course of the chorda tympani nerve, (c) as well as the presence of support structures due to the inverted vat polymerisation printing technique.

The fixation of the ossicular chain may potentially be overcome by designing hinges at the ossicular joints, or by printing these joints with a more flexible material from the ossicles using material jetting techniques. These techniques may also be used to depict the intra-osseous course of the chorda tympani nerve in a different colour or to create support structures that are soluble. The performance of the material jetting material on drilling would also need to be assessed.

These methods, together with the conduct of a formal study with more participants comparing the efficacy of the model in simulating surgical procedures to cadaveric or live surgery, augmenting the existing literature on the efficacy of temporal bone simulation models in improving training outcomes [13], would form future directions of this work.

## Conclusion

We review the literature on the features and development methods as well as describe a systematic development workflow for 3D-printed temporal bones. With these models, the training of otolaryngological surgeons can be enhanced and made more accessible with 3D printing.

### Supplementary Information

Supplementary Material 1. [2, 8, 10, 14–27]

## Data Availability

No datasets were generated or analysed during the current study.

## Abbreviations

3D: 3-Dimensional
CAD: Computer-Aided Design
CI: Cochlear Implant
CT: Computed Tomography
DfAM: Design for Additive Manufacturing
DICOM: Digital Imaging and Communication
FFF: Fused Filament Fabrication
HU: Hounsfield Unit
IPA: Isopropyl Alcohol
keV: Kilo-electron-volt
mAs: milliampere-seconds
OSHA: Occupational Safety and Health Administration
