# Supplementary material for: Developing a production workflow for 3D-printed temporal bone surgical simulators
Source: 3D Print Med. 2024 May 30;10:16. doi: 10.1186/s41205-024-00218-x (PMC11138071; doi:10.1186/s41205-024-00218-x)
Supplement: Supplementary file 1 — Supplementary Material 1. [2, 8, 10, 14–27] [file 41205_2024_218_MOESM1_ESM.docx]

SUPPLEMENTAL INFORMATION

Table S1: Summary table of the literature review.

| **First Author** | **Year** | **Key finding** | **Model Material** | **Manufacturing Workflow** | **Conceptual Contribution** | **Limitations of Study** |  |
| --- | --- | --- | --- | --- | --- | --- | --- |
| Bakhos D et al. [14] | 2010 | Inverted vat polymerisation can be used to develop a good anatomic model of TB | White resin | 3D Doctor for imaging processing Magics for file repair before conversion Inverted vat polymerisation was used to print models | Use of inverted vat polymerisation to develop TB models Dye was used to colour soft tissue structures | Stapes malleus and incus were absent in multiple prototypes Facial nerve canal was dehiscent which allowed leakage of dye |  |
| Haffner M et al. [15] | 2013 | PETG outperformed PLA, ABS, PC and Nylon for realistic representation and haptic feedback of a TB model. | ABS, PC, PLA, PETG and Nylon | 3D Slicer processed DICOM data Cura prepared the 3D print Ultimaker Extender 3 was used to print the models | Comparison of multiple FDM thermoplastics revealed PETG had the best performance. | Fine anatomy of the TB models was not discussed. |  |
| Cafino R et al. [16] | 2014 | Photopolymer and PETG are suitable materials to be used for dissection, with resin models showing more accuracy for anatomical representation. | PETG, Simubone and Resin. | Materialize Mimics converted DICOM to STL Meshmixer was used to create a base Cura prepared the 3D print Ultimaker 3 and Elegoo Saturn 2 were used for printing | Testing of a new bone-like material, Simubone in the context of surgical simulation of TB. | All models were not suitable for labyrinthectomy. |  |
| Mowry SE et al. [17] | 2015 | Development of a TB model on a desktop printer. | ABS | OsirX converted DICOM to STL Netfabb used to slice STL Makerbot 2x 3D printer printed models | Use of desktop printer to develop inexpensive TB models | Mastoid air cells unsuccessfully recreated using ABS |  |
| Da Cruz MJ et al. [18] | 2015 | synthetic temporal bone has a high degree of face and content validity | Cast powder | Phacon models were used. | Micro-CT data was used for higher fidelity Colour was added to differentiate structures Effective method of modelling the mastoid air cells via material removal was described | Tympanic and round window membrane not modelled Mobility of the ossicular chain not simulated Facial nerve and bony fallopian canal interface not simulated Dura and sigmoid sinus colour contrast not achieved. |  |
| Chenebaux M et al. [19] | 2016 | Resin-based model is an alternative for cadaveric TB for drilling technique training. | White resin (Somos). | 3D Doctor used for image processing Materialize Mimics used for preparing STL files Inverted vat polymerisation was used to print models | Use of hand painting to add colour in the model. | Facial nerve colour was excessively vivid Identifying the posterior semicircular canal was difficult. Ossicular chain was rigid. |  |
| Wanibuchi et al. [20] | 2016 | Separating the model into two halves allowed for augmentation of internal structures. | Polyamide nylon | Aquilion 64 was used to obtain DICOM data  Materialize Mimics used for preparing STL files  SLS was used to print models | Model was made in two pieces to process the mastoid air cells and dye semicircular canals | No limitations were mentioned. |  |
| Frithioff A et al. [21] | 2017 | In-house production of cost-effective 3D printed models is possible.  Laybrick was the best material compared. | Wood-/Metal-/Copper-/Concrete-filled PLA, PVA +, Nylon, ABS, Laybrick | OpenEar library models were used Solidworks was used to add a mounting block Ender 3 Pro was used to print the model | Use of open-source data to develop a TB model | Chorda tympani nerve was not represented Lateral semicircular canal and ossicles lacked visual cues. |  |
| Rose AS et al. [2] | 2018 | Multi-material TB models have potential benefits in surgical training. | Photopolymer | Materialize Mimics converted DICOM to STL files Objet350 Connex was used to print the models | Using a blend of multiple materials to achieve colour scaling and varying physical characteristics. | Cochlear labyrinth was not completely patent |  |
| Freiser et al. [10] | 2018 | Procedures performed on models made from PLA, ABS and resin were safe by OSHA standards. | ABS, PLA, Resin | FlashForge Creator Pro was used to print the PLA and ABS model  Formlabs Form 2 was used to print the resin model | VOC testing showed that drilling models were safe. |  |  |
| Chien WW et al. [22] | 2019 | 3D printed models were anatomically realistic compared to cadaveric temporal bones | Cast powder | Phacon models | Validation of commercially available Phacon models. | Ossicular chain was not well represented |  |
| McMillan et al. [23] | 2020 | FLW, photopolymer and PC were superior materials for simulators with FLW being the most cost-effective. | ABS, Photopolymer, PC and Resin | Materialize Mimics converted DICOM to STL Makerbot 2x (ABS) Objet350 Connex3 (photopolymer) FDM-Fortus (PC) Formlabs Form 2 (FLW, FLB) | Comparison of multiple materials and printing processes. Use of drainage holes for Polyjet prints to wash away supports. | Mastoid air cells were difficult to replicate in FDM models. |  |
| Gadaleta DJ et al. [24] | 2020 | TB models were anatomically accurate and cost-effective | PLA | 3D Slicer processed DICOM data  Meshmixer optimized file for 3D printing Ultimaker 3 printed models | Use of colour to differentiate Internal structures of interest | Facial nerve canal, Ossicular chain were not well represented |  |
| Mowry SE et al. [8] | 2020 | Models printed in standard resin material with inverted vat polymerisation scored highest in the evaluation. TB models have potential benefit in surgical training and standardized testing. | PC, Powder, Visijet PXL Calcium Sulfate Hemihydrate, Wood/PLA, and multiple resins. | Multiple models were sent to the team. | Large-scale comparison of multiple TB models. | Resin has glare under the microscope. Spaces of the middle ear and mastoid air cells were filled with resin. |  |
| Chauvelot et al [25] | 2020 | 3D printed resin TB models can quantitatively reproduce original anatomy | Resin | Blender was used to segment the DICOM file  Different resins with varying hardness were used to produce TB models | Use of engineering techniques to validate anatomical accuracy of model | No simulation was performed on the models. |  |
| Takahashi K et al. [26] | 2021 | The 3D printed TB model was sufficiently practical for use in surgical training. | Plaster powder and coloured binders | ZedView was used to develop STL models Drainage holes were added to remove excess material Projet 460 Plus was used to print the model | Drainage holes were introduced to remove excess materials from air-containing spaces and soft tissue structures were coloured differently | Stapes, tympanic sinus, and mastoid air cells were unsatisfactory. |  |
| Freiser ME et al. [27] | 2024 | Patient-specific 3D printed models provide an anatomically accurate and favorable tool for preparing for middle cranial fossa surgery | Inverted vat polymerisation | Discovery CT750 HD imaged cadaveric TB 3D Slicer developed STL files Meshmixer and Blender were used to refine model Formlabs Forms 2 used to print models. | Developing a model to allow for MCF surgery training. | Ossicles were fused Bone density and colour differences were not replicated |  |
| Legend: 3D Manufacturing Format, STL; Acrylonitrile Butadiene Styrene, ABS; Digital Imaging and Communications in Medicine, DICOM; Formlabs Blue Resin, FLB; Formlabs White Resin, FLW; Fused-Deposition Modelling, FDM; Middle Cranial Fossa, MCF; Not applicable, N.A.; Polycarbonate, PC; Polyethylene terephthalate glycol, PETG; Polylactic acid, PLA; Polyvinyl alcohol, PVA; Occupational Safety and Health Administration, OSHA; Temporal bone, TB; Volatile organic compounds, VOC. | | | | | | | |
